# Longitudinal changes in health behaviours and body weight among Swedish school children - associations with age, gender and parental education – the SCIP school cohort

**DOI:** 10.1186/1471-2458-14-640

**Published:** 2014-06-23

**Authors:** Liselotte Schäfer Elinder, Nelleke Heinemans, Zangin Zeebari, Emma Patterson

**Affiliations:** 1 Department of Public Health Sciences, Karolinska Institutet, Tomtebodavägen 18A, Stockholm 171 77, Sweden; 2 Centre for Epidemiology and Community Medicine, Stockholm County Council, Box 1497, Solna 171 29, Sweden

**Keywords:** BMI, Body weight status, Children, Diet, Gender, Longitudinal changes, Socioeconomic status, Physical activity

## Abstract

**Background:**

In order to develop health promotion initiatives it is important to identify at what age gender and socioeconomic inequalities in health-related behaviours emerge. The aim of this longitudinal study was to analyse how health-related behaviours and weight status differed by age-group, gender, family socio-economic status and over time in three cohorts of school children.

**Methods:**

All children in grades 2, 4 and 7 in a Swedish semi-urban municipality were invited to participate (n = 1,359) of which 813 (60%) consented. At baseline and after 2 years a health questionnaire was answered by all children. Height and weight was measured. Fourteen outcomes were analysed. The main and interaction effects of time, gender and parental educational level on the health-related behaviours, weight status and body mass index standard deviation score (BMIsds) were analysed by the Weighted Least Squares method for categorical repeated measures and Analysis of Variance.

**Results:**

Nine of 12 health behaviours deteriorated over the two years: consumption of breakfast and lunch, vegetables and fruit, intake of sweetened drinks, TV viewing, club membership, being outdoors, and school recess activity; two behaviours were unchanged: intake of sweets, and active transport. Only sports participation increased with time. Girls consumed more vegetables, less sweetened drinks, performed less sports, were less physically active during recess, and had lower BMIsds, compared to boys. Those with more highly educated parents had more favourable or similar behaviours compared to those with less educated parents in 10 out of 12 health behaviours, the only exception being intake of sweets and being outdoors, and had lower BMIsds.

**Conclusions:**

This study adds to our knowledge regarding the temporal development of health behaviours and weight status in school children. Differences with regard to gender and socioeconomic status were seen already at a young age. These results contribute to our understanding of several important determinants of obesity and chronic diseases and may inform future interventions regarding how to decrease gender and social inequalities in health.

## Background

In order to maintain good health throughout life, and to reduce the risk of chronic disease, health-related behaviours like dietary habits and physical activity are of crucial importance [1]. Even though it is never too late to change behaviour, it is preferable to have a healthy diet and be physically active from an early age in order to promote optimal growth and development. In Sweden [2], as in many countries in Europe [3], children’s diets contain too much saturated fat, sugar and salt and too little fruit and vegetables. Findings from the Health Behaviour of School-aged Children (HBSC) study suggest consistent inequalities associated with parental socioeconomic status (SES) in self-reported health, psychosomatic symptoms, physical activity and aspects of eating habits at both the individual and country level [4] and also in overweight and obesity in most European countries [5]. With regard to physical activity, socioeconomic differences are not consistently found in children under 12, but are more likely to emerge during adolescence [6,7]. Socioeconomic inequalities have consistently been found in Sweden [8-10], especially with regard to obesity, which is two to six times higher in the lowest socioeconomic group compared to the highest [10].

Factors that have been linked to obesity in children and young adults include an irregular meal pattern [11], in particular omission of breakfast [12-14], a low intake of fruit and vegetables [15], high-fat/energy-dense foods and sweetened beverages [16], a low level of moderate-to-vigorous physical activity [13,17], and sedentary behaviour. In particular, the evidence suggests that daily TV viewing in excess of 2 hours has been associated with reduced physical and psychosocial health, and higher BMI [18,19]. Furthermore, studies have demonstrated that with increasing age a deterioration is seen in physical activity levels [20-22] and dietary habits [23] and that mother’s educational level is a positive predictor of retaining a healthy eating pattern [24].

In order to develop effective health promotion initiatives it is important to identify at what age socioeconomic and gender inequalities in health-related behaviours start to become established. Cohort studies with a long follow-up period and frequent measurements provide valuable information on temporal changes but such studies are rare, as they take a long time to conduct, are expensive, and the drop-out rate increases markedly as time goes by [25]. A more feasible approach is to study multiple cohorts spanning the primary school years with fewer data collection waves. The SCIP-school study was a whole-school intervention study with the aim of improving eating habits, physical activity levels and promoting a healthy body weight in children aged 6-16. The study population consisted of all children in a semi-urban municipality in grades 2, 4 and 7 who were measured at baseline in 2009 and again in 2011. Positive and significant impacts were noted in health promotion practices in the schools in the areas of physical activity, mental health and eating habits as a result of the 2-year intervention. However, at the student level no significant intervention effects were seen for the main outcomes [26].

The aim of this study was to analyse how health-related behaviours and weight status differed by age-group, gender and family socio-economic status, as well as over time in the three age cohorts of children.

## Method

### Study design, schools and recruitment

The SCIP-school study was a 2-year intervention study with nine intervention and nine comparison schools, conducted in the municipality of Österåker in Stockholm County. This middle class municipality has approximately 39,000 inhabitants and is characterized as semi-urban according to Statistics Sweden. In 2009, all children in grades 2, 4 and 7 in the municipality were invited to participate (n = 1,359). In total 813 (60%) of the children and their parents consented: 307 children in grade 2 (“grade 2 cohort”), 300 children in grade 4 (“grade 4 cohort”), and 206 children in grade 7 (“grade 7 cohort”). The proportion of parents with a higher education (>12 years) in our sample was 64%, compared to 54% in the whole population of this municipality. Overweight and obesity among all grade 4 children in the municipality was 21% compared to 19.4% in our sample. At follow-up, data on body weight was available for 795 children, whereas questionnaires were available from 694 children. The highest parental educational level was used as a measure of family socioeconomic status and dichotomised into higher education (>12 years) or no higher education (12 or less years of education). This information was obtained at follow-up. Since no significant intervention effects at student level were found after two years of intervention [26], we combined the data from the intervention and comparison schools. Written informed consent was obtained from all participating children and their parents. Ethical permission for this study was obtained from the Regional Ethical Review Board in Stockholm County No. 2009/280-31/5.

### Outcomes

#### Anthropometry

Height and weight was measured in a standardized way by the school nurse or by the research team. BMI was calculated (weight (kg)/height (m^2^)). Normal weight, overweight and obesity were defined using cut-off points according to the International Obesity Task Force [27]. BMI standard deviation scores (BMIsds), adjusted for age and sex, were obtained based on the normal Swedish population reference curves [28].

#### Health behaviours

The diet and physical activity questionaires were tested for reliability and relative validity [26]. The diet questions were tested against a 7-day food diary in 55 grade 4 and 38 grade 7 students and Cohen’s weighted kappa was calculated to measure agreement between the two methods. Values were obtained ranging from 0.24-0.54 in grade 4 and from 0.35-0.88 in grade 7. To test reliability, a test-retest analysis was performed on questionnaires administered three days apart and kappa values of 0.17-0.59 for grade 4 and 0.65-0.96 for grade 7 students were obtained. The physical activity questionnaire was tested for validity by comparing it to data from an accelerometer worn for 7 days in 46 grade 4 and 38 grade 7 students. We found 64% agreement in the same category for grade 4 and 58% for grade 7 children. At baseline (April-May 2009) and after 2 years (April-May 2011) a health questionnaire was sent home to all children, to be answered with the help of a parent if necessary. The diet questions covered behaviors such as frequency of breakfast and lunch consumption, intake of fruit, vegetables, soft drinks, and sweets. The response options were on a four-point ordinal scale, ranging from “never” to “every school day” for breakfast and lunch. The other diet questions had five response options from “twice per day” to “seldom”. For this analysis the responses were dichotomized so that they corresponded to adhering/not adhering to current dietary recommendations; i.e. the cut-points for the dichotomization were set at “every school day” for breakfast and lunch, “twice a day” for both fruit and vegetables, and “no more than twice per week” for soft drinks and sweets.

The physical activity question about leisure time exercise/sports had four response options, ranging from “every day” to “not at all”. Active commuting was reported as the number of days per week the children walked or biked to school. Physical activity during recess was reported as the number of days they were physically active during recess. For each of these variables, a dichotomous variable was created with answers of up to “less than three days per week” indicating low physical activity. One further question assessed whether the child was a member of a sports club or any other club with the response options “yes” or “no”.

TV-viewing was assessed as hours watching TV on school days, using four predefined categories, ranging from “less than 1 hour a day”, “1-3 hours a day”, “3-6 hours a day”, and “more than 6 hours a day”. A dichotomous variable was computed for “at most three hours per day” and “more than three hours per day”.

### Data analysis

The main and interaction effects of age-group, parental educational level, gender and time (2 years) on the health-related behaviours and anthropometry of the students were analysed. For categorical repeated measures, the Weighted Least Squares (WLS) method [29] was used for modelling the marginal logits of the dichotomous outcomes without missing values at baseline or follow-up. For the continuous repeated measure (BMIsds), analysis of variance (ANOVA) was used. With the WLS method, the signs of the parameter estimates and their Wald goodness-of-fit statistics (analogous in functionality to ANOVA) were used to detect the direction and the significance of each factor on the outcomes. The analysis was carried out in three steps:

Step 1: All data were entered into the analysis in order to be able to assess the main and interaction effects of cohort, gender, parental educational level and time on each outcome.

Step 2: As for step 1 plus stratification for cohort.

Step 3: As for step 2 plus stratification for gender.

The results of the WLS analysis were confirmed using the Generalized Estimating Equations (GEE) method [30] to again model the marginal logits. The GEE method handles missing data, and therefore its estimates were compared to those of the WLS method. For children who had no follow-up data, an analysis was conducted to detect potential systematic differences with respect to baseline values of all outcomes. A chi-square test was used for proportions, and in case of small cell counts Fischer’s exact test was used. The Mann–Whitney *U* test was used to assess differences regarding weight status. For BMIsds as the outcome, a *t*-test was used. Analyses were performed using the statistical package SAS 9.3. The significance level was set at 5%.

## Results

Participant characteristics, health behaviours, and weight status, are shown in Table 1 per cohort, for girls and boys separately. All health behaviour outcomes were expressed so that a higher percentage is desirable from the point of view of optimal health. The number of individuals at follow up is the number of children who had an observation on at least one of the outcomes. The proportion of missing data at follow up for each outcome variable was between 5.6% (BMIsds for boys in grade 7) and 20.4% (intake of fruit for girls in grade 7).

**Table 1:** Characteristics of participants and health behaviours at baseline and follow up by gender and by cohort

	**Grade 2 cohort**		**Grade 4 cohort**		**Grade 7 cohort**	
	**Baseline N = 307**	**Follow-up N = 299**	**Baseline N = 300**	**Follow-up N = 294**	**Baseline N = 206**	**Follow-up N = 202**
**Girls**	N = 151	N = 148	N = 157	N = 155	N = 98	N = 97
Age (years ± SE)	8.74 ± 0.03	10.74 ± 0.03	10.81 ± 0.03	12.81 ± 0.03	13.90 ± 0.03	15.90 ± 0.03
Parental education high (%)	-	69.6	-	65.0	-	56.1
Eats breakfast every school day (%)	96.6	98.4	91.7	89.1	84.5	67.5
Eats lunch every school day (%)	96.5	91.3	87.2	76.6	65.6	73.5
Eats vegetables at least twice a day (%)	44.4	40.0	35.7	24.8	28.9	32.5
Eats fruit at least twice a day (%)	34.0	22.2	24.8	15.9	12.4	16.9
Eats sweets at most twice a week (%)	81.4	78.4	76.1	82.4	73.2	74.7
Drinks sweetened drinks at most twice a week (%)	84.1	81.8	81.9	77.5	86.6	78.3
Watches TV at most three hours every school day (%)	100.0	95.2	98.0	92.8	94.9	89.2
Is member of a club (%)	85.5	84.1	85.9	80.4	78.1	62.7
Participates in sports at least three times a week (%)	31.9	54.4	50.3	57.3	56.7	51.2
Spends at least 30 minutes outdoors every school day (%)	93.1	76.2	89.0	81.0	79.8	65.1
Walks or bikes to school at least three days a week (%)	64.8	78.6	77.1	70.4	72.6	60.5
Is physically active during breaks at least 3 days a week (%)	70.6	54.0	71.3	20.4	14.6	3.6
Obese (%)	3.3	0.7	3.2	1.9	3.1	1.0
Overweight/obese (%)	15.9	14.6	18.5	12.1	14.3	12.2
BMIsds (mean ± SE)	0.27 ± 0.09	0.07 ± 0.10	0.30 ± 0.08	0.33 ± 0.08	0.22 ± 0.12	0.19 ± 0.10
**Boys**	N = 156	N = 151	N = 143	N = 139	N = 108	N = 105
Age (years ± SE)	8.77 ± 0.02	10.77 ± 0.02	10.80 ± 0.02	12.80 ± 0.02	13.91 ± 0.03	15.91 ± 0.03
Parental education high (%)	-	65.5	-	62.0	-	60.2
Eats breakfast every school day (%)	97.4	95.6	94.3	86.9	86.8	75.0
Eats lunch every school day (%)	97.4	85.3	88.7	82.0	63.2	76.1
Eats vegetables at least twice a day (%)	31.1	25.2	36.9	18.0	21.7	20.5
Eats fruit at least twice a day (%)	29.1	14.1	23.9	11.5	14.2	9.3
Eats sweets at most twice a week (%)	79.3	83.2	78.2	80.3	74.5	69.3
Drinks sweetened drinks at most twice a week (%)	81.3	79.4	72.5	70.5	58.5	48.9
Watches TV at most three hours every school day (%)	100.0	97.1	98.6	95.0	92.3	87.5
Is member of a club (%)	86.7	82.5	90.9	76.9	77.4	65.9
Participates in sports at least three times a week (%)	56.7	66.4	69.7	64.5	78.1	62.5
Spends at least 30 minutes outdoors every school day (%)	92.7	86.8	89.7	76.0	83.0	78.4
Walks or bikes to school at least three days a week (%)	54.7	76.7	83.6	61.0	78.3	56.3
Is physically active during recess at least 3 days a week (%)	80.3	81.6	86.4	40.5	48.1	19.5
Obese (%)	2.6	5.1	4.9	5.6	1.9	1.9
Overweight/obese (%)	20.5	18.6	20.3	18.2	18.5	15.7
BMIsds (mean ± SE)	0.53 ± 0.10	0.61 ± 0.11	0.55 ± 0.10	0.41 ± 0.10	0.33 ± 0.10	0.27 ± 0.10

For each outcome, baseline values were compared between children who did and did not provide data on that outcome at follow-up. Those with a higher body weight at baseline were significantly (p < 0.05) more likely to have missing values at follow-up. Likewise, club membership was significantly lower at baseline among those who had no values at follow up. For all other outcomes no significant differences were found at baseline for those with missing values at follow up versus those with no missing value for that outcome (data not shown).

Table 2 shows the effects of age-cohort, gender, parental education and time on all outcomes (corresponding to step 1 of the analysis described in Method).

**Table 2:** Associations between cohort, gender, parental educational level and time and behavioural and weight outcomes

	**Grade 4 cohort**^**a**^	**Grade 7 cohort**^**a**^	**Gender**^**b**^	**Parental educational level**^**c**^	**Time**^**d**^
Eats breakfast every school day	0	–	0	+	–
Eats lunch every school day	0	–	0	+	–
Eats vegetables at least twice a day	0	0	+	+	–
Eats fruit at least twice a day	0	–	0	0	–
Eats sweets at most twice a week	0	–	0	–	0
Drinks sweetened drinks at most twice a week	0	–	+	+	–
Watches TV at most three hours every school day	0	–	0	0	–
Is member of a club	+	–	0	+	–
Participates in sports at least three times a week	0	+	–	+	+
Spends at least 30 minutes outdoors every school day	0	–	0	–	–
Walks or bikes to school at least three days a week	0	0	0	+	0
Is physically active during recess at least 3 days a week	0	–	–	0	–
Overweight/obese	0	0	0	0	–
BMIsds	0	0	–	–	0

^a^Compared to grade 2 cohort.

^b^Girls compared to boys. The sign + (–) stands for higher (lower) proportion among girls, when other factors (cohort, parental education and time) are adjusted for.

^c^Higher compared to lower parental education. The sign + (–) stands for higher (lower) proportion among children with high parental education, when other factors (cohort, gender and time) are adjusted for.

^d^Time at follow up compared to time at baseline. The sign + (–) stands for higher (lower) proportion among children at follow-up, when other factors (cohort, gender and parental education) are adjusted for.

Healthy behaviours were observed more frequently in the two youngest cohorts compared to the oldest cohort for most outcomes. The exception was sports participation, which was higher in the oldest cohort. Girls consumed more vegetables, less sweetened drinks, performed less sports, were less physically active during recess, and had lower BMIsds compared to boys. Children with more highly educated parents had more favourable or similar behaviours compared to those with less educated parents in 10 out of 12 health behaviours, the only exceptions being intake of sweets and being outdoors, and also had lower BMIsds. Nine of the 12 health behaviours deteriorated over the two years: consumption of breakfast and lunch, vegetables and fruit, intake of sweetened drinks, TV viewing, club membership, being outdoors, and recess activity. Two behaviours were unchanged: intake of sweets, and active transport. Only sports participation increased with time. The percentage of overweight and obesity decreased with time while BMIsds was unchanged.

**Figure 1:**
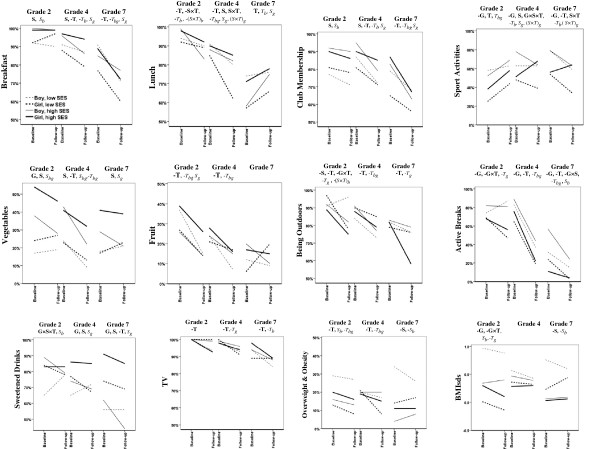
**Developments in 12 outcomes in the three cohorts over a two year period including effect of gender and parental education.** The symbols **G, S** and **T** stand for Gender, Socioeconomic status/parental educational level, and Time, respectively. The symbols × and – indicate an interaction and negative direction of effect, respectively. An *italic* symbol indicates a factor with significant effect after stratification for gender. Subscript symbols *b* and *g* stand for boys and girls, respectively.

Figure 1 shows 12 of the outcomes stratified according to cohort, gender and parental education (corresponding to steps 2 and 3 in Method). In step 2 of the analysis (stratification according to cohort) a bold symbol stands for a factor with significant effect on the outcome. The symbols G, S and T stand for Gender, Socioeconomic status/parental educational level, and Time, respectively, and the symbols × and – indicate an interaction and negative direction of effect, respectively. In step 3 of the analysis (cohort and gender stratification) an *italic* symbol stands for a factor with significant effect. Subscript symbols *b* and *g* stand for strata of boys and girls, respectively. A test of significance was based on the WLS estimates except for some cases where the GEE estimates agreed better with the graphs, namely effect of SES on breakfast and lunch for girls in grade 7. The graphs in Figure 1 should be interpreted as follows, using breakfast as an example. In the grade 2 cohort, there is a significant difference in breakfast consumption between SES groups (S), but when stratified for gender it appears that this difference is mainly due to a SES-effect in boys only (*S*_*b*_). In the grade 4 cohort we see a significant effect of SES (S), as well as a decrease over time (-T, from 2009 to 2011). When stratified for gender, the effect of time is significant for boys (-*T*_*b*_) but not for girls, while the effect of SES is only significant for girls (*S*_*g*_). In the grade 7 cohort we see a decrease over time (-T), which is significant for both boys and girls (-*T*_*bg*_), and a significant effect of SES in girls only (*S*_*g*_).

## Discussion

The aim of this study was to analyse how health-related behaviours and weight status differed by gender, family socio-economic status and over a 2-year period in three cohorts of school children in grades 2, 4 and 7. The main findings were that most health behaviours deteriorated between each cohort and also over time within each cohort, even in the youngest cohort, when children are 8 years of age. Furthermore, gender differences were most pronounced with regard to physical activity levels, which were generally higher in boys. Socioeconomic differences were found for several outcomes both regarding diet, physical activity and BMIsds, where children with higher educated parents had better outcomes.

### Diet and BMI

The prevalence of overweight and obesity for 10-year old boys and girls (the grade 4 cohort) found in this study corresponds well to the prevalence found in a larger study conducted in the greater Stockholm area by Sundblom et al. in 2003 [9]; 20.5% vs. 20.3% for boys, and 18.5 vs. 19.2% for girls. This is another indication that the overweight and obesity prevalence seems to be stabilizing as suggested by Rokholm et al. [31,32]. In a more recent nationally representative sample of 7-9 year old children from Sweden the prevalence of overweight and obesity in semi-urban areas (corresponding to our study) was reported to be 14.3% among girls and 21.1% among boys [10]. This is similar to the prevalence in the youngest cohort (grade 2, 8-year olds) in our study, where it was 15.9% for girls and 20.5% for boys.

In agreement with our finding of an inverse effect of parental educational level on BMIsds, these authors also found significant differences in obesity prevalence according to mother’s and father’s educational level [10]. Sundblom et al. also found higher overweight and obesity prevalence in geographical areas in Stockholm where the population had a low socioeconomic status [9]. At European level, lower levels of overweight have also been found in children of more highly educated parents than in children of less educated parents [3].

According to the socio-ecological model for health, the reasons for this consistent social gradient can be found at many levels. At the family level, and in particular for pre-teenage children, parental permissiveness, role modeling and encouragement are suggested to play important roles for children’s health-related behaviours [25]. These findings highlight the need for parental support, sensitive to parental educational level, as an intervention strategy to prevent overweight and obesity. The observed decrease in overweight and obesity prevalence over time in the three cohorts should be taken with some caution due to the fact that children with a higher BMI were slightly more prone to attrition than normal weight children.

Considering the higher BMIsds values among boys in general and among children with less educated parents all the behavioural dietary outcomes, except for sweets, were in the expected direction. With regard to breakfast, around 90% of the children in grade 2 ate breakfast every school day. However, breakfast consumption started to decline from grade 4 and showed a further strong decline from grade 7 to 9 with children of less educated parents showing a tendency towards a sharper decline. Skipping breakfast has a negative impact on academic achievement by adversely affecting cognition and attendance [14] even if the effects are more apparent in children whose nutritional status is compromised [33]. Furthermore, according to a recent 18-year longitudinal study, daily breakfast consumption is associated with a 20% reduced risk of a spectrum of metabolic conditions such as abdominal obesity, obesity, metabolic syndrome, hypertension and type 2-diabetes [12]. One significant predictor of children’s breakfast consumption is having breakfast together with the parents who act as role models [25]. We found a similar picture for daily lunch consumption, which started to decline already in the grade 2 cohort. In particular girls with less educated parents showed a dramatic decline - 85% ate school lunch every day in grade 4 compared to 60% in grade 6, despite the fact that in Sweden free school lunches are provided to all children in grades 1-9, regardless of parental income. Interestingly, lunch consumption increased again after grade 7 in all groups, which could be explained by the concurrent decline in breakfast consumption, which would result in hunger at lunchtime. Skipping meals is prospectively associated with a larger BMI increase in both men and women [11]. Therefore, encouraging all children to eat breakfast and lunch daily has the potential to lead to lower BMI and lower social inequalities in health.

The intake of fruit and vegetables was generally low among the children in our study. Only 35% of girls ate vegetables two or more times every day, and among boys this percentage was around 20%. Results from the UK have shown similar results, where the vegetable and fruit intake of 7-year old children was far below the recommended intake, with girls eating more than boys [34]. Factors associated with intake in the UK study were parental level of education, parental consumption, parental rules and child’s preferences. As shown by Bere et al. [35] the gender difference can to a large extent be explained by a lower preference for vegetables by boys, while the perceived accessibility at home can explain the major part of the difference between socioeconomic groups [36]. In adults, vegetable intake is probably the dietary behaviour which differs most by gender and socioeconomic status. Only 13% of women and 5% of men reach the recommended intake of 500 grams per day in Sweden according to the most recent national dietary survey [37]. Taken together, fruit and vegetable intake deserves much greater public health attention given the high importance for health and prevention of chronic diseases [1]. Increasing the consumption among boys, men and low socioeconomic groups has the potential to contribute to lowering of social inequalities in health. Interventions, ideally including parental support, should start at the earliest possible age in order to establish preferences in young boys.

Sweets consumption did not show any strong associations with age, gender or parental educational level in our study. In contrast, the intake of sweetened beverages increased with age, was higher in boys than girls and also showed a clear inverse association with parental education. Considering the evidence regarding the effects of sweetened drinks on unhealthy weight gain [38] it is possible that this item contributed to the higher BMI levels among boys and lower socioeconomic groups in this sample of children. A literature review has shown that determinants of intake of sweetened beverages are availability at home and in school, parental consumption and parenting style, as well as household socioeconomic status [39], in agreement with our results. Future interventions should be very clear in their messages sent to families with regard to sweetened beverages in a way that reaches those groups with the highest consumption.

### Physical activity

Boys participated to a higher extent in sports and were more active during recess compared to girls in our study. This confirms previous Swedish studies, where physical activity was assessed with objective methods, which showed higher activity levels among boys [10,22]. A study by Nyberg et al. in Sweden showed that the difference in physical activity levels between girls and boys was most pronounced during school time, as opposed to leisure time, and that the decline in physical activity started as early as the age of 6 [22]. We noted a strong decline with increasing age in both active recess and being outdoors, supporting these findings. Among girls this trend was observed as early as in the grade 2 cohort (8 years at baseline), whereas in boys it declined from grade 4. Being outdoors showed the same trend. A longitudinal study from the USA showed a reduction of 50% and 66%, for boys and girls respectively, in moderate to vigorous activity between the ages of 9 and 15 [21]. Another study from the UK showed decreasing activity levels in both boys and girls from age 7 to 9 [20]. In our study, active transport increased between grade 2 and 4 where it peaked but then declined in the older age cohorts. It is well known that being outdoors is associated with higher activity levels in children [40] and a study from Sweden has shown that a high-quality outdoor environment is associated with several health aspects in children such as leaner body, longer night-sleep and better well-being [41]. Therefore, activity-promoting school yards should receive high priority as a measure to increase children’s physical activity that could potentially decrease social inequalities in health, as all children would benefit equally.

Club membership, sports participation and active transport were higher in children from families with higher education, suggesting the role of parents in enabling such behaviours. Active travel to school is an important source of physical activity [42] associated with healthier body weight and cardiorespiratory fitness in youth and should be encouraged. No association with parental education was found for any other physical activity behaviour. A large cross-sectional study in 10-12 year old children from seven European countries showed an association between parental educational and physical activity levels only in two and three countries for girls and boys, respectively [43]. An Australian study following two cohorts aged 5-6 and 10-12 at baseline for three years from 2001 to 2004 also found decreasing levels of physical activity with age, but no strong evidence that mother’s education predicted, cross-sectionally or longitudinally, physical activity or sedentary behaviours [6]. Since socioeconomic inequalities in physical activity are consistently observed among adults, the authors speculated that differences start to occur after the age of 12.

### Sedentary behaviour

Evidence is accumulating that time spent sitting is a health risk behaviour for adults [44], but in children and young people the health effects are less clear. However, there is data to suggest that sedentary behavior is associated with unhealthy dietary behaviours in children, adolescents and adults [45]. Systematic reviews have concluded that there is strong evidence for associations between screen-based sedentary behaviour for more than two hours per day and higher weight status in children [18,46], as well as sleep problems, musculoskeletal pain and depression in girls [18]. In more detailed analysis using quantile regression, positive associations between screen time and changes in the upper tail of the BMI distribution only were observed independently of levels of physical activity [47]. In our study we found that TV-viewing gradually increased with age: almost all children in grade 2 watched less than three hours per day, but in grade 9 15% watched TV for more than three hours a day, similar to results from the UK for the age group 12 to 16 [48]. However, no significant differences in TV-viewing with regard to gender or parental education were found. The real number of screen-based hours in this study was likely to be much higher since computer-time was not included, which means that a much higher proportion of children are at risk from excessive sedentary behaviour. One factor known to affect TV-viewing duration is whether parents impose rules or restrictions [39].

### Strengths and limitations

The consent rate in this study was on the low side (60%), but not exceptionally so considering active consent was sought from both participating children and their parents. One strength of this study is that every child in this semi-urban middle class municipality was invited to participate, reducing selection bias. The level of education among parents in our sample was higher compared to Sweden as a whole and also higher than in the general population in the municipality, but the level of overweight and obesity among 10-year olds was very similar. This increases the likelihood that the results are generalizable to a major part of the Swedish population. Furthermore, the children were followed longitudinally, their weight and height was measured and the questionnaires used had reasonable validity. The most apparent limitation is the fact that all health behaviours were self-reported. Recall validity is generally lower in younger children compared to older, but among grade 4 and 7 students validity was at least moderate. Furthermore, recall data is prone to error, including “desirability bias” which would likely have resulted in reporting of more positive behaviours, and possibly to a less steep socioeconomic gradient, than the “true” reality.

## Conclusion

This study adds to our knowledge regarding the temporal development of health behaviours and weight status in school children. Differences with regard to gender and socioeconomic status were seen already at a young age. These results contribute to our understanding of several important determinants of obesity and chronic diseases and may inform future interventions regarding how to decrease gender and social inequalities in health.

## Competing interests

The authors declare that they have no competing interests.

## Authors’ contributions

LSE conceived of the study, participated in its design and coordination and drafted the manuscript. NH participated in data collection, interpretation of results and drafting of the manuscript. ZZ designed and carried out the statistical analysis and participated in the drafting of the manuscript. EP participated in the interpretation of results and drafting of the manuscript. All authors read and approved the final manuscript.

## Pre-publication history

The pre-publication history for this paper can be accessed here:

http://www.biomedcentral.com/1471-2458/14/640/prepub
